# Efficacy and safety of delafloxacin, ceftaroline, ceftobiprole, and tigecycline for the empiric treatment of acute bacterial skin and skin structure infections: A network meta-analysis of randomized controlled trials

**DOI:** 10.1016/j.jsps.2021.12.007

**Published:** 2022-01-01

**Authors:** Abdullah A. Alhifany, Nisrin Bifari, Yasser Alatawi, Saad U. Malik, Thamer A. Almangour, Ali F. Altebainawi, Thamir M. Alshammari, Amal F. Alotaibi, Ahmad J. Mahrous, Fahad S. Alshehri, Ejaz Cheema

**Affiliations:** a College of Pharmacy, Riyadh Elm University, Riyadh, Saudi Arabia; b Department of Pharmacy Practice, College of Pharmacy, University of Tabuk, Tabuk, Saudi Arabia; c Department of Hematology and Oncology, College of Medicine, University of Arizona, Tucson, USA; d Department of Clinical Pharmacy, College of Pharmacy, King Saud University, P.O. Box 2457, Riyadh 11451, Saudi Arabia; e Pharmaceutical Care Services, King Salman Specialist Hospital, Hail Health Cluster, Hail, Saudi Arabia; f Medication Safety Research Chair, King Saud University, Riyadh, Saudi Arabia; g Department of Pharmacology and Toxicology, College of Pharmacy, Umm Al-Qura University, Makkah, Saudi Arabia; h School of Pharmacy, University of Management and Technology, Lahore, Pakistan

**Keywords:** Empiric, Treatment, Acute, Bacterial, Skin, Infection, Review

## Abstract

**Background:**

This review aimed to conduct an indirect comparison using a Bayesian network meta-analysis of randomized controlled trials (RCTs) to compare the efficacy and safety of delafloxacin versus other single antibiotic regimens for the empiric treatment of Acute Bacterial Skin and Skin Structure Infections.

**Method:**

A systematic search with no start date restrictions was conducted. The Cochrane Risk of Bias tool was used to assess the quality of included RCTs.

**Results:**

Of the 577 studies initially identified, nine RCTs were included in the review. The network meta-analysis showed that ceftaroline, ceftobiprole, delafloxacin and tigecycline had similar efficacy in the indirect comparisons [Ceftaroline Odds Ratio (OR) = 1.2, 95% Crl = 0.46–3.6), ceftobiprole (OR = 1.3, 95% Crl = 0.34–3.0) and tigecycline (OR = 0.96, 95% Crl = 0.30–2.9)]. However, the ranking plot for the intention to treat (ITT) population showed that delafloxacin had a probability of 80.8% to be ranked first followed by ceftobiprole (13.1%). The analysis of the overall adverse events showed that ceftaroline (OR = 0.88, 95% Crl = 0.65–1.2), ceftobiprole (OR = 1.1, 95% Crl = 0.69–2.0), delafloxacin (OR = 0.88, 95% Crl = 0.57–1.3) and tigecycline (OR = 1.4, 95% Crl = 0.88–2.2) had similar safety profiles.

**Conclusion:**

Delafloxacin did not show any statistically significant differences when compared to ceftaroline, ceftobiprole, and tigecycline in terms of efficacy and safety. However, the surface under the cumulative ranking curve (SUCRA) probability ranked delafloxacin as the first option for the ITT population.

## 1 Introduction

Acute bacterial skin and skin structure infection (ABSSSI) is a skin-infection with lesion size of at least 75 cubic centimeters (cm2) such as cellulitis, wound infection and major cutaneous abscesses (Pollack Jr et al., 2015). Treatment of ABSSSI depends on the type and severity of the infection and should aim to cover both Methicillin-Sensitive Staphylococcus aureus (MSSA) and Methicillin-Resistant Staphylococcus aureus (MRSA) (Esposito et al., 2017). However, in recent years, there has been an increase in the prevalence of gram-negative and anaerobic organisms causing ABSSSI (Bassetti et al., 2013, Guillamet and Kollef, 2016, Itani et al., 2011). It has been reported that many patients with complicated skin and soft-tissue infections (SSTI) initially received inappropriate empiric antibiotics that subsequently led to treatment failure and infection deterioration due to the lack of gram-negative coverage (Edelsberg et al., 2009, Zilberberg et al., 2010, Zilberberg et al., 2014). Therefore, the provision of appropriate empirical antibiotic therapy that covers both gram-positive and gram-negative bacteria is critical, particularly in complicated SSTI such as diabetic foot infections, burn wound infections and gas gangrene due to the suspected involvement of polymicrobial organisms or gram-negative bacteria (Edelsberg et al., 2009, Zilberberg et al., 2010, Zilberberg et al., 2014).

The Infectious Disease Society of America (IDSA) guidelines recommend treating ABSSSI with broad-spectrum empiric antibiotics that have MRSA, gram-negative and anaerobic coverage such as vancomycin, linezolid or daptomycin plus piperacillin-tazobactam or a carbapenem. However, vancomycin plus aztreonam is the most commonly used antibiotic combination in clinical trials (Stevens et al., 2014). Nevertheless, the frequent dosing and monitoring requirements of vancomycin and daptomycin to prevent nephrotoxicity and/or creatinine phosphokinase (CPK) accumulation (Ye ZK, Li C, & SD., 2014) together with the black box warning of linezolid to cause retinopathy and the necessity to maintain peripheral intravenous (IV) access during hospital admission are among some of the factors that warrant the need for alternative therapeutic options (Almangour et al., 2017, Almangour et al., 2019). Therefore, many new single agents with MRSA and gram-negative coverage, oral options and/or good safety profile have been developed to serve as potential alternatives to the standard combination therapy in the treatment of ABSSSI. Some of these agents include ceftaroline, ceftobiprole, tigecycline, and the recent United States (US) Food and Drug Administration (FDA) approved delafloxacin (Breedt et al., 2005, Corey et al., 2010, Dryden et al., 2016, Noel et al., 2008, O’Riordan et al., 2018, O'Riordan et al., 2015, Pullman et al., 2017, Sacchidanand et al., 2005, Talbot et al., 2007, Wilcox et al., 2010).

With the exception of one phase 2 clinical trial that has compared delafloxacin with tigecycline (O'Riordan et al., 2015), there are no head-to-head randomized controlled trials (RCTs) that have compared delafloxacin with other single agents. Hence, the main objective is to conduct an indirect comparison using a Bayesian network meta-analysis (NMA) of RCTs to compare the efficacy and safety of delafloxacin versus other single antibiotic regimens for the empiric treatment of ABSSSI.

## 2 Methods

The systematic review and network meta-analysis were conducted according to the Preferred Reporting Items for Systematic Reviews and Meta-Analyses (PRISMA) extension statement for network meta-analyses (Moher, Liberati, Tetzlaff, Altman, & Group, 2010).

A systematic search with no start date restrictions was conducted in February 2020 in four databases, including PubMed/Medline, Embase, Scopus and Clinicaltrials.gov. Studies were retrieved up to 27th February 2020. Searches were undertaken using medical subject headings (MeSH) terms and free keywords including adult, comparison, safety, efficacy, single, double, antimicrobial OR antibiotic, empiric OR empirical, acute bacterial skin, skin structure, infection, randomized controlled trial and treatment. Searches were conducted using the Patients, Intervention, Comparator, Outcome, and Study design (PICOS) strategy (Table 1).

**Table 1:** PICOS strategy for clinical evidence of antibiotics used in the management of skin and soft tissue infections.

**PICOS**	**Clinical review**
Population	Adults with skin infections (e.g., cellulitis, skin and soft tissue infections, complicated skin and skin structure infections, acute skin and skin tissue infections).
Intervention	A monotherapy parenteral or oral antibiotic that covers gram-positive
	(MRSA and MSSA) and gram-negative pathogens.
Comparator	Standard-of-care dual therapy that covers gram-positive (MRSA and MSSA) and gram-negative pathogens
Outcome	1. Response, resolution, or clinical cure
	2. Adverse drug reaction
Study design	Published or unpublished randomized controlled trials of any size and duration

### 2.1 Inclusion and exclusion criteria

RCTs of any size and duration that compared the efficacy and safety of any single-antimicrobial agent versus standard-of-care treatment (i.e., dual-antimicrobial agents) in treating adult patients with ABSSSI were eligible for inclusion. Non-RCT studies, were excluded from the analysis. In addition, studies that included pediatric patients or infections caused by gram-positive bacteria only were also excluded.

The efficacy outcome considered for this review was based on the US-FDA and the European Medicines Agency (EMA) guidelines for ABSSSI (EMA., 2010, Fda, 2013). The efficacy outcome analyzed was the clinical success defined as either cure (complete resolution) or improved (some symptoms remained), with no additional need for antibiotics for clinically evaluable (CE) patients (who completed activities as defined in the protocol) or for intent-to-treat (ITT) populations (all patients randomized) at follow up (FU), which is generally 7–14 days after the completion of the treatment. The safety outcomes assessed were the overall adverse events (AEs), serious adverse events, and any related AEs that led to the discontinuation of treatment or death.

### 2.2 Data extraction, risk of bias and quality assessment

Two reviewers (NB and SM) independently reviewed the titles and abstracts. Articles that met the inclusion criteria were retrieved as full papers and these two reviewers checked each paper for inclusion. Any differences were agreed through discussion or resolved by a third reviewer (AA). Reviewer AA independently extracted data from included studies.

The Cochrane Risk of Bias tool was used to assess the quality of included RCTs including randomization, allocation concealment, blinding of participants, reporting of incomplete outcome data, selective reporting and any other bias (Higgins, 2008). Other sources of bias explored included cross-contamination between study groups, recruitment of participants from a selected population and non-compliance with the study protocol. For each included study, risk of bias graphs and risk of bias summary were generated (Higgins, 2008).

### 2.3 Statistical analysis

A network meta-analysis was conducted to compare the efficacy and safety of different single-antibiotic agents for the empiric treatments of ABSSSI. Analyses were conducted using a Bayesian approach with Markov chain Monte Carlo estimation using GEMTC. The treatment effects were presented as Odds Ratio (OR) with values greater than one indicating a superior outcome for any of the antimicrobial agents compared to the comparators. A random effect model was utilized to estimate the relative effect to account for the heterogeneity of the included studies. The value of burn-in iteration and inference iteration were set at 5,000 and 40,000, respectively. Model convergence was considered suitable when the value of the potential scale reduction factor was less than 1.05 and the density plot was smooth with regular shape. Results were expressed as odd ratios (ORs) with 95% credible intervals (CrI).

## 3 Results

**Fig. 1:**
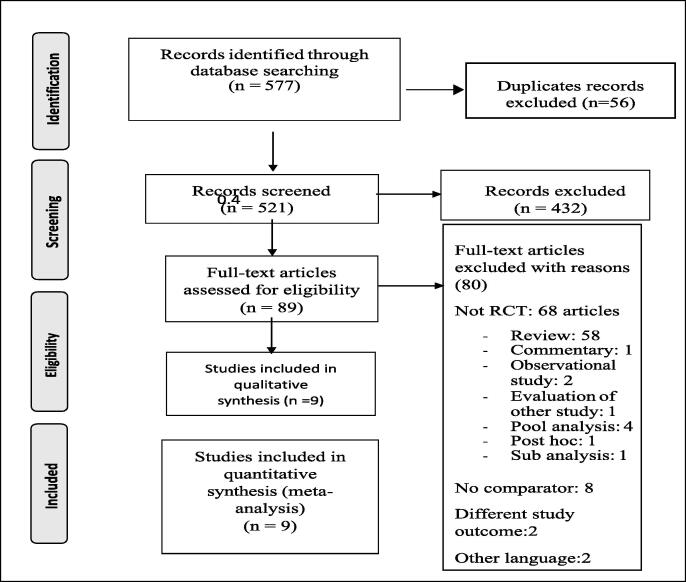
Study selection process using preferred reporting items for systematic reviews and meta-analyses (PRISMA).

A total of 577 articles were identified through initial searches (see Fig. 1). Fifty-six duplicates were removed and 521 articles were reviewed at the abstract level, with 432 irrelevant abstracts removed. After removing abstracts not meeting the inclusion criteria, 89 full-text articles were reviewed. Of these, 80 articles did not meet the inclusion criteria. Reasons for exclusion included non-RCTs, articles with no comparator, articles with different study outcome and articles in non-English. Nine articles met the study inclusion criteria and contributed to the review and network meta-analysis (Breedt et al., 2005, Corey et al., 2010, Dryden et al., 2016, Noel et al., 2008, O’Riordan et al., 2018, O'Riordan et al., 2015, Pullman et al., 2017, Sacchidanand et al., 2005, Talbot et al., 2007, Wilcox et al., 2010).

**Fig. 2:**
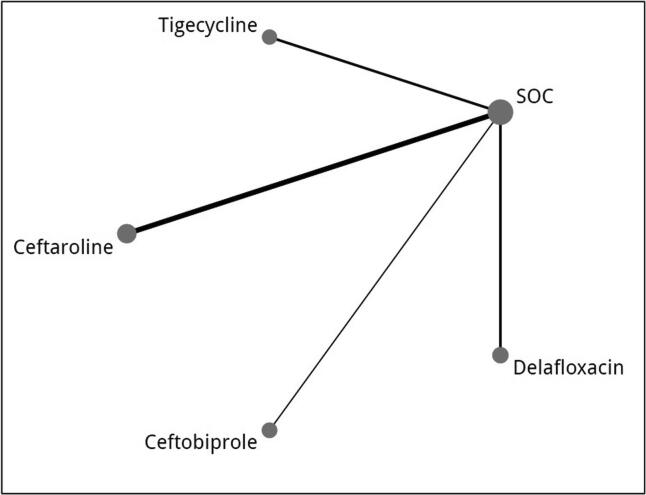
Network plot of included studies. Node represent each drug and the size of each node indicates the number of included participants. Lines show the direct comparisons and the line thickness represent the number of studies included in each comparison. SOC: Standard of care (i.e., dual treatment).

All studies were published between 2005 and 2018 (see Table 2 for study characteristics and Table 3 for the efficacy outcomes reported in the included studies). 5633 participants were included in the final analysis (1266 ceftaroline, 538 tigecycline, 754 delafloxacin, 547 ceftobiprole, 2247 vancomycin/aztreonam, and 281 vancomycin/ceftazidime). Four studies investigated ceftaroline, two investigated tigecycline, and two investigated delafloxacin compared to vancomycin plus aztreonam. One study investigated ceftobiprole compared to vancomycin plus ceftazidime (see Fig. 2 for the network plot of the included studies).

**Table 2:** Characteristics of included studies.

**Author/publication year**	**Study design**	**Mean age (Intervention/Comparator)**	**Intervention**	**Comparator**	**Treatment Duration**
Talbot et al. 2007	Multinational, Phase 2 single-blinded RCT	41.6/44	Ceftaroline: 600 mg every 12 h IV (n = 67)	Vancomycin (1 g every 12 h) + aztreonam (1 g every 8 h) then de- escalate according to culture result. (n: 33)	7 to 14 days. Up to 21 days in severe infection
Dryden et al. 2016	Multinational, Phase 3 double-blinded RCT	52.6/53.6	Ceftaroline fosamil IV (600 mg every 8 h) (n = 514)	Vancomycin (15 mg/kg every12 h) plus Aztreonam (1 g every 8 h)., Aztreonam could be discontinued after ≥ 3 days if no Gram-negative bacteria were identified (n = 258)	5–14 days
Ralph Corey et al. 2010	Multinational, Phase 3 double-blinded RCT	47.2/49.2	Ceftaroline: 600 mg every 12 h (n = 353)	Vancomycin 1 g plus aztreonam 1 g every 12 h (n = 349)	5–14 days
Wilcox et al. 2010	Multinational, Phase 3 double-blinded RCT	47.8/47.5	Ceftaroline: 600 mg every 12 h (n = 348)	Vancomycin 1 g plus aztreonam 1 g every 12 h (n = 346)	5–14 days.
Sacchidanand et al. 2005	Multinational, Phase 3 double-blinded RCT	49.4/48.4	Tigecycline 100 mg initial dose, followed by 50 mg twice daily (n = 295)	Vancomycin 1 g BID + Aztreonam 2 g BID (n = 288)	Up to 14 days.
Breedt et al. 2005	Multinational, Phase 3 double- blinded RCT	48.8/50.1	Tigecycline 100 mg initial dose, followed by 50 mg twice daily (n = 275)	Vancomycin 1 g BID + Aztreonam 2 g BID, Aztreonam could be discontinued after 48 h, according to the investigator’s clinical judgment. (n = 271)	Up to 14 days.
O'Riordanet et al. 2018	Multinational, Phase 3 double- blinded RCT	51.2/50.2	Delfloxacin: if CrCl> 29 ml/ min: delafloxacin: 300 mg IV BID for 6 doses followed by 450 mg tablet BID, If CrCl < 29: 200 mg IV BID for all doses (n=423)	Vanc+ AZT: If CrCl > 29 : Vancomycin 15 mg / kg BID, Aztreonam: 2 g BID./ If CrCl < 29: Vancomycin adjusted dose and aztronam 1g BID (n=427)	5–14 days
Pullman et al. 2017	Multinational, Phase 3 double-blinded RCT	46.3/45.3	Delafloxacin: 300mg IV BID. (n=331)	Vancomycin 15mg/kg + Aztreonam 2 g every 12h which was discontinued once baseline cultures did not reveal Gram negative bacteria (n=329)	5–14 days
Noel et al. 2008	Multinational, Phase 3 double-blinded RCT	52.9/51.9	Ceftobiprole: 500 mg every 8h. (n=547)	Vancomycin + Ceftazidime: started 1 g of Vanc BID then adjusted according to the level + 1g of ceftazidime TID (n=281)	7–14 days

**Table 3:** Efficacy outcomes (Clinical Success) at follow up of the included studies.

**Authors/ publication year**	**Delafloxacine 300 mg**		**Tigecycline**		**Ceftaroline**		**Ceftobiprole**		**Vancomycin/Aztreonam**		**Vancomycin/Ceftazidime**	
	Clinical success events / Total population treated											
	CE	ITT	CE	ITT	CE	ITT	CE	ITT	CE	ITT	CE	ITT
Talbot et al. 2007					59/ 61	59/ 67			24/ 27	26/32		
Dryden et al. 2016					342/ 395	396/ 506			180/ 211	202/ 255		
Ralph Corey et al. 2010					288/ 316	304/ 351			280/ 300	297/ 347		
Wilcox et al. 2010					271/ 294	291/ 342			269/ 292	289/ 338		
Sacchidanand et al. 2005			165/ 199	209/ 277					163/ 198	200/ 260		
Breedt et al. 2005			200/ 223	220/ 261					201/ 213	225/ 259		
O'Riordanet et al. 2018	340/ 353	369/ 423							319/ 329	362/ 427		
Pullman et al. 2017	233/ 240	270/ 331							238/ 244	274/ 329		
Noel et al. 2008							439/ 485	448/ 547			220/ 244	227/ 281

### 3.1 Quality assessment

The quality of included RCTs was variable with all studies reporting random sequence generation. Only two (22%) of the nine studies reported details of allocation concealment. It was unclear in the remaining seven studies whether they had used adequate allocation concealment. (see Fig. 3A for risk of bias graph and 3B for the risk of bias summary).

**Fig. 3A:**
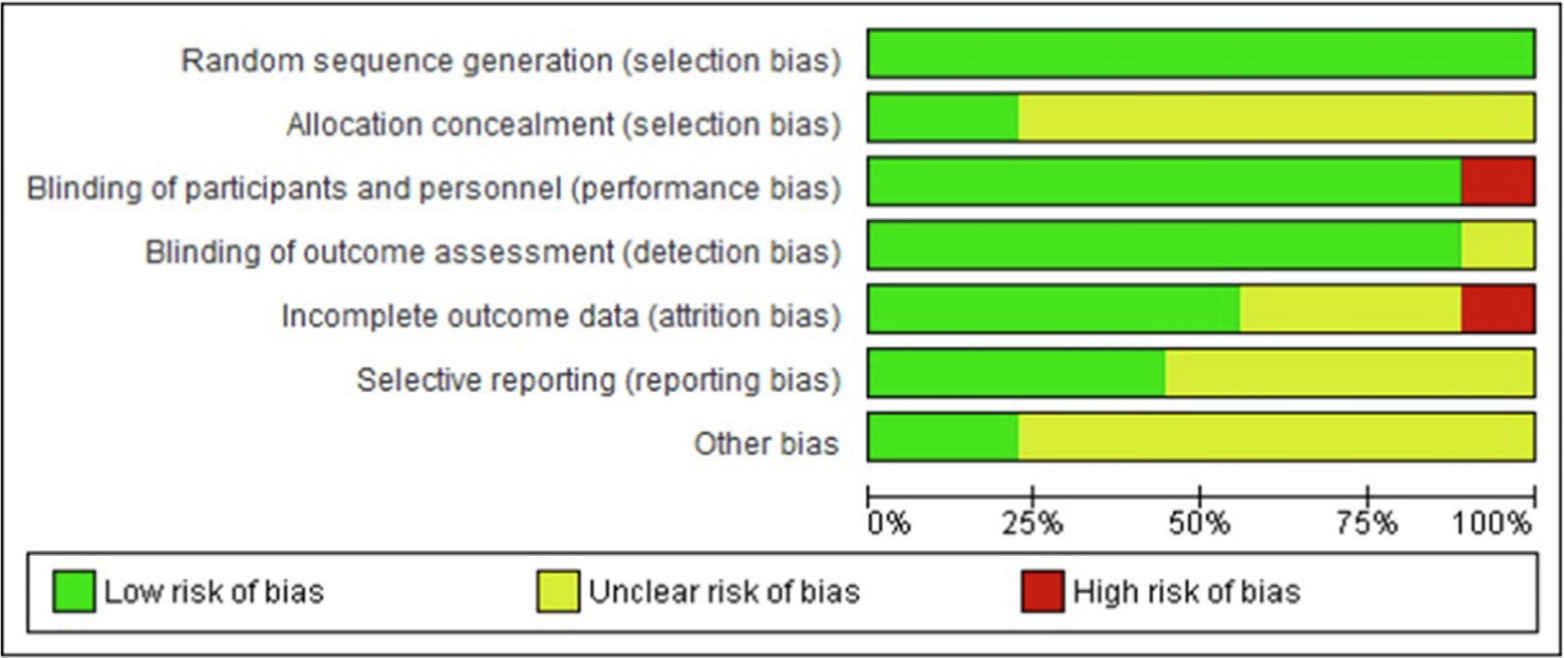
Risk of bias graph: review authors' judgements about each risk of bias item presented as percentages across all included studies.

**Fig. 3B:**
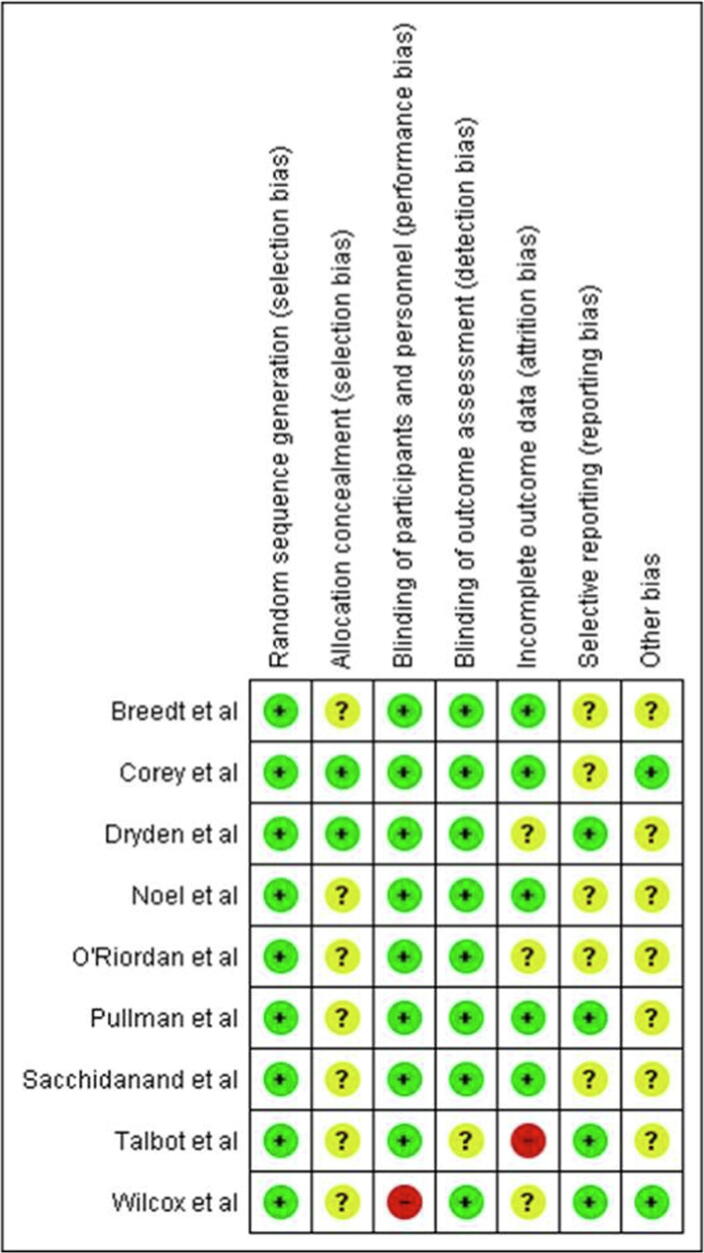
Risk of bias summary: review authors' judgements about each risk of bias item for each included study.

### 3.2 Efficacy

**Fig. 4:**
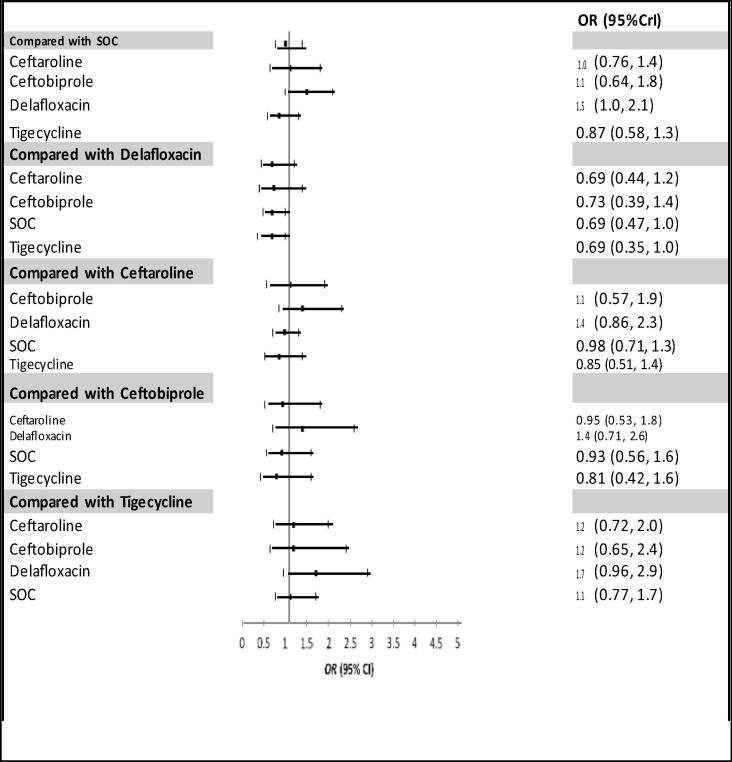
Forest Plot of included studies in the network meta-analysis for the random effect model for the Intent-to-treat (ITT) population. Square shows the relative effect (odd ratio: OR) for each drug with 95% credible interval (CrI).

**Fig. 5:**
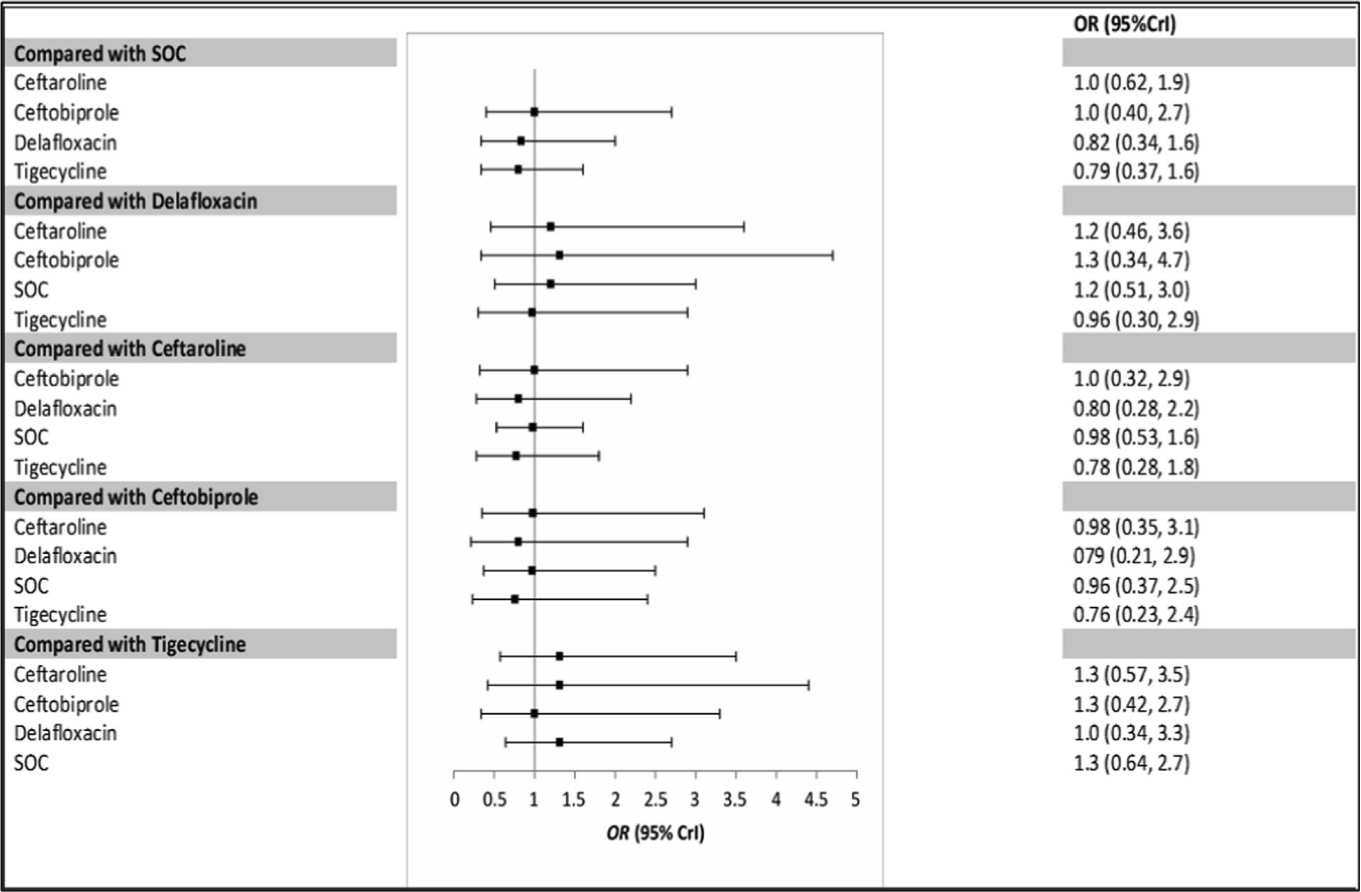
Forest Plot of included studies in the network meta-analysis for the random effect model for clinical evaluable population (CE). Square shows the relative effect (odd ratio: OR) for each drug with 95% credible interval (CrI).

The random effect models converged well with the potential scale reduction factor values of less than 1.05, and the density plots were smooth with regular shape. Fig. 4, Fig. 5 represent the forest plots of the Bayesian network meta-analysis (NMA) for the clinical success for the ITT populations (Fig. 4) and CE populations (Fig. 5). The analysis showed that single-antimicrobial agents did not produce significantly different results compared to standard-of-care treatments with ceftaroline (OR = 1.0, 95% Crl = 0.76–1.4), ceftobiprole (OR = 1.1, 95% Crl = 0.64–1.8), delafloxacin (OR = 1.5, 95% Crl = 1.0–2.1) and tigecycline (OR = 0.87, 95% Crl = 0.58–1.3) showing no statistically significant differences compared to the dual standard of care regimen for the ITT population. Similar results were also found for the CE population.

**Fig. 6:**
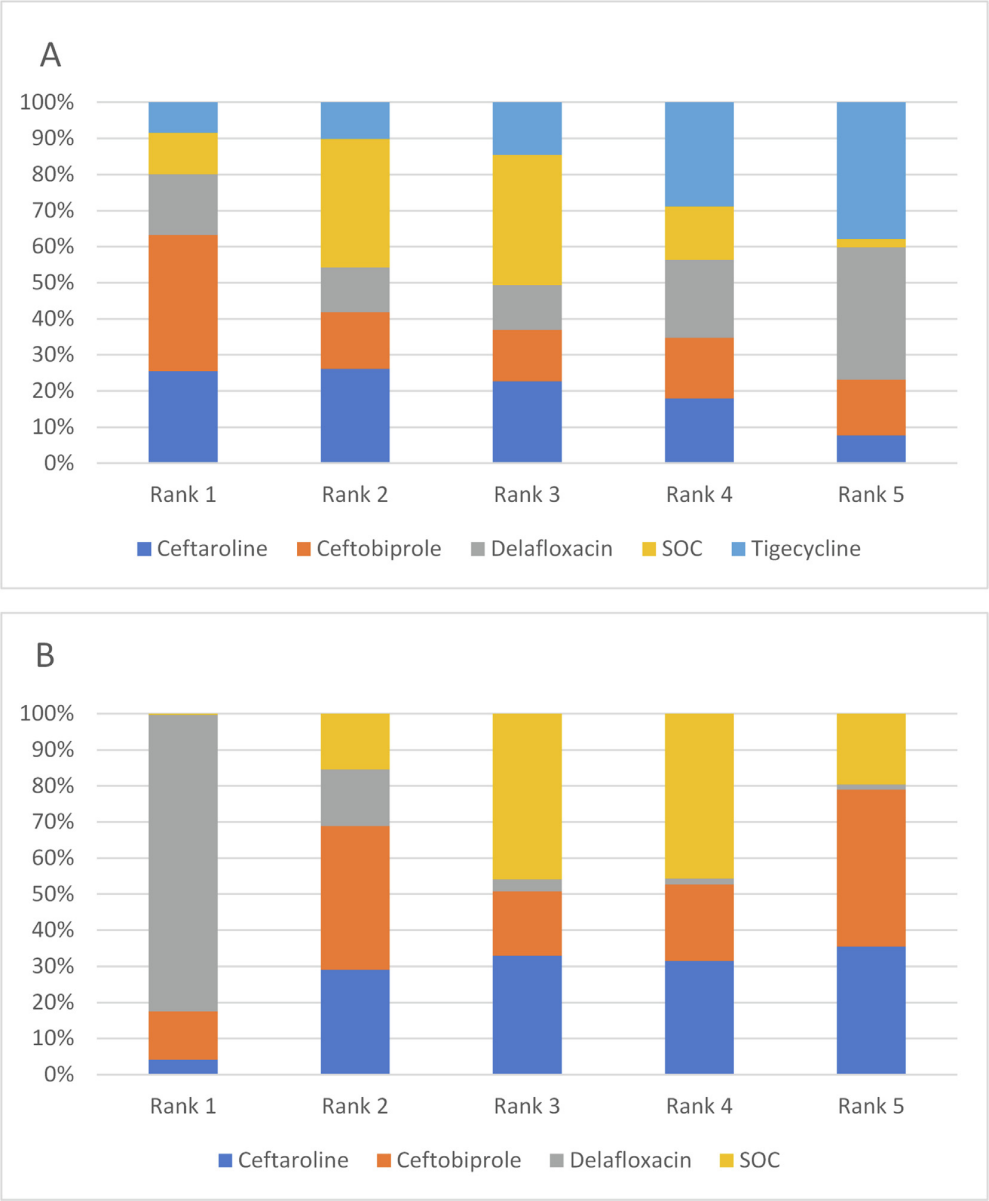
Ranking probability plot for the network meta-analysis using surface under the cumulative ranking curve (SUCRA) for each endpoint. Endpoints are A) Clinical Evaluable population; B) intent-to-treat population.

The results also showed that ceftaroline, ceftobiprole, delafloxacin and tigecycline had similar efficacy in the indirect NMA comparisons [ceftaroline (OR = 1.2, 95% Crl = 0.46–3.6), ceftobiprole (OR = 1.3, 95% Crl = 0.34–3.0) and tigecycline (OR = 0.96, 95% Crl = 0.30–2.9). Furthermore, ceftobiprole (OR = 1.0, 95% Crl = 0.32–2.9) and tigecycline (OR = 0.78, 95% Crl = 0.28–1.8) had similar efficacy compared to ceftaroline. On the other hand, the ranking plot of the NMA for ITT population showed that delafloxacin had a probability of 80.8% to be ranked first, followed by ceftobiprole (13.1%). However, the analysis for the CE population showed a higher probability for ceftobiprole to be ranked first (37.7%) followed by ceftaroline (25.5%) (Fig. 6).

### 3.3 Adverse events

**Fig. 7:**
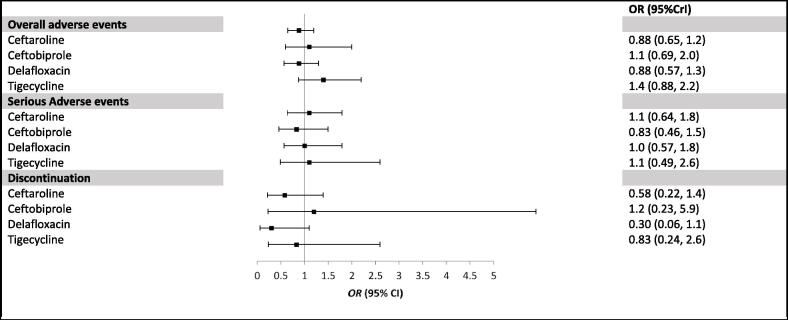
Forest Plot of included studies in the network meta-analysis for the random effect model of safety-related endpoints. Square shows the relative effect (odd ratio: OR) for each drug with 95% credible interval (CrI).

The majority of the studies reported data on the overall and serious adverse events as well as information related to the discontinuation of antimicrobial agents. Overall, single-antimicrobial agents had similar safety profiles compared to the standards of care treatment. The analysis of the overall adverse events showed that ceftaroline (OR = 0.88, 95% Crl = 0.65–1.2), ceftobiprole (OR = 1.1, 95% Crl = 0.69–2.0), delafloxacin (OR = 0.88, 95% Crl = 0.57–1.3) and tigecycline (OR = 1.4, 95% Crl = 0.88–2.2) were not significantly different compared to the standard of care. Similar results were found for serious adverse events and discontinuation of treatment when using single-antimicrobial agents compared with the standard of care treatment (Fig. 7).

## 4 Discussion

To authors’ knowledge, this is the first network meta-analysis that has indirectly compared delafloxacin with other single antibiotics indicated for the treatment of ABSSSI. The findings of this study suggest that delafloxacin did not show any statistically significant difference in terms of efficacy and safety when compared with ceftaroline, ceftobiprole, and tigecycline. However, the SUCRA ranking probability ranked delafloxacin as the first option for the ITT population. Despite the variability among the included studies, this network meta-analysis comparison between the single antibiotics and the standard of care dual regimens showed consistent results with the direct head-to-head clinical trials.

Delafloxacin is a new fluoroquinolone antibiotic that has been approved by the FDA in 2017 for the treatment of ABSSSI. In vitro studies have suggested that it has good activity against both gram-positive (including MRSA) and gram-negative (including P. aeruginosa) bacteria (Pfaller et al., 2017, Remy et al., 2012). This activity offers a monotherapy option for the treatment of SSTI caused by polymicrobial organisms such as diabetic foot and burn wound infections. Furthermore, it has both intravenous and oral formulations that allow patients to switch to an oral route that consequently shortens the length of hospital stay and allows physicians to treat ABSSSI in the outpatient settings (O'Riordan et al., 2018). In comparison to older fluoroquinolones, delafloxacin does not appear to have an effect on QT intervals or photosensitivity reactions (Dawe et al., 2018, Litwin et al., 2015). A phase-2 RCT that compared two doses of delafloxacin with tigecycline did not find any difference in the clinical cure rates and microbiologic eradication (O'Riordan et al., 2015). Furthermore, the most common adverse events (AEs) reported for delafloxacin were gastrointestinally related which intensified with higher doses coupled with mild central nervous system (CNS) events (O’Riordan et al., 2018, O'Riordan et al., 2015, Pullman et al., 2017). Nevertheless, delafloxacin should still be used with caution due to the FDA warnings including the increased risk of tendonitis, peripheral neuropathy and CNS disturbances, although these adverse events have not been reported in clinical trials involving delafloxacin (Tillotson, 2016).

Tigecycline has been associated with GI problems (nausea, vomiting), risk of acute pancreatitis, and has limited activity against Pseudomonas aeruginosa (Gales and Jones, 2000, Hung et al., 2009). Similarly, ceftaroline is well known for diarrhea, nausea and rash (Corey et al., 2010, Wilcox et al., 2010). Ceftobiprole, on the other hand, has a broad spectrum of activity that covers Gram-positive including MRSA, Gram-negatives including susceptible Pseudomonas species and some anaerobes which makes it a good option for the treatment of complicated skin infections, yet it is only marketed in Europe and has not received US FDA approval (Dauner, Nelson, & Taketa, 2010).

This review has some limitations. The quality of the included RCTs was variable, with more than half of the studies not reporting using adequate allocation concealment. Although rigorous and systematic, the reviewers did not include unindexed and unpublished research. Nevertheless, it is the first study to provide an indirect comparison in terms of efficacy and safety between delafloxacin and other single antibiotic regimens (ceftaroline, ceftobiprole, and tigecycline). Even though no difference has been reported, a ranking probability has been provided to help the readers pick the best option that meets the population of interest.

## 5 Conclusion

Delafloxacin did not show any statistically significant differences when compared to ceftaroline, ceftobiprole, and tigecycline in terms of efficacy and safety. However, the SUCRA ranking probability ranked delafloxacin as the first option for the ITT population.

## Declaration of Competing Interest

The authors declare that they have no known competing financial interests or personal relationships that could have appeared to influence the work reported in this paper.
